# Comparison of rapid solvent extraction systems for the GC–MS/MS characterization of polycyclic aromatic hydrocarbons in aged, contaminated soil

**DOI:** 10.1016/j.mex.2016.04.007

**Published:** 2016-04-26

**Authors:** Nagalakshmi Haleyur, Esmaeil Shahsavari, Abdulatif A. Mansur, Eman Koshlaf, Paul D. Morrison, A. Mark Osborn, Andrew S. Ball

**Affiliations:** a Centre for Environmental Sustainability and Remediation, School of Science, RMIT University, Bundoora, Melbourne, VIC 3083, Australia; b Environmental and Natural Resources Engineering, Faculty of Engineering, Azawia University, Libya

**Keywords:** Comparison of rapid solvent extraction systems for the GC–MS/MS characterization of polycyclic aromatic hydrocarbons in aged, contaminated soil, GC–MS/MS, PAHs, Solvent extraction, Bioremediation, Soil contamination, Weathered soils

## Abstract

Polycyclic aromatic hydrocarbons (PAHs) are a major class of organic hydrocarbons with high molecular weight that originate from both natural and anthropogenic sources. Sixteen PAHs are included in the U.S Environmental Protection agency list of priority pollutants due to their mutagenic, carcinogenic, toxic and teratogenic properties. In this study, the development and optimization of a simplified and rapid solvent extraction for the characterisation of 16 USEPA priority poly aromatic hydrocarbons (PAHs) in aged contaminated soils was established with subsequent analysis by GC–MS/MS.

• Five different extraction solvent systems: dichloromethane: acetone, chloroform: methanol, dichloromethane, acetone: hexane and hexane were assessed in terms of their ability to extract PAHs from aged PAH-contaminated soils.
• Highest PAH concentrations were extracted using acetone: hexane and chloroform: methanol. Given the greater toxicity associated with chloroform: methanol, acetone: hexane appears the best choice of solvent extraction system.
• This protocol enables efficient extraction of PAHs from aged weathered soils.

## Graphical abstract

**Figure:**
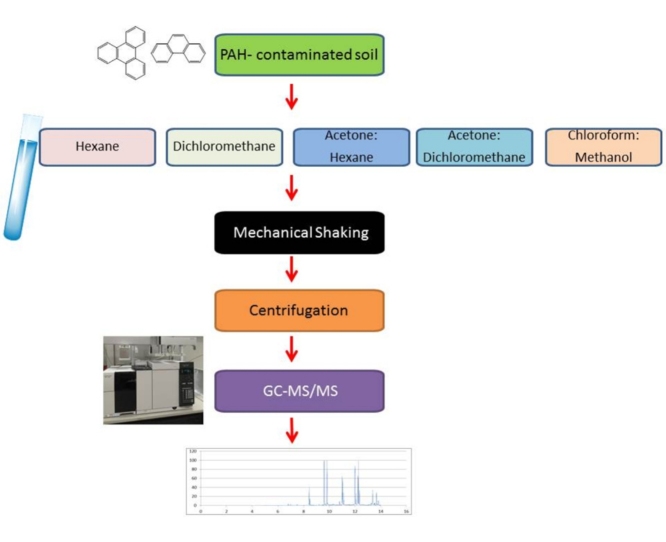

## Protocol background

The widespread use of petroleum hydrocarbons and mismanagement of hydrocarbon waste and other by-products from various industries has led to the extensive contamination of terrestrial and aquatic environments through spills and leakages [1]. The identification and quantification of PAHs in aged weathered soils is particularly difficult. The amount of PAHs extracted declines rapidly with the age of the soil mainly due to sequestration and strong adherence to soil organic matter [2]. The sorption of hydrophobic organic contaminants to the soil matrix also decreases the rate and extent of biodegradation [3]. Weathered PAH contaminated soil normally contains a recalcitrant fraction of compounds composed of high molecular weight hydrocarbons which could not be degraded by indigenous microorganisms [4]. It is important from a risk management perspective that the technique and the solvent used to extract the PAHs from the soil matrix are efficient and reflects the true concentration of PAHs.

Due to the current limitations associated with traditional extraction methods, including being instrumentally demanding, time consuming, tedious, expensive and often involving large volumes of toxic solvents new approaches are being devised for the extraction of PAHs to counter these drawbacks. In addition, several extract clean-up steps are also required upstream of gas chromatography (GC) to protect GC columns prior to analysis [5]. GC coupled with tandem mass spectrometry is widely used for the analysis of PAHs due to its high specificity and sensitivity [6], [7], [8], [9], [10], [11], [12], [13], [14], [15]. The aim of this study was to assess the effectiveness of rapid solvent extraction systems in combination with GC–MS/MS, using a multiple monitoring mode (MRM) [12] database to quantify PAHs (specifically 16 US EPA priority pollutants) from weathered contaminated soils. To the best of the authors’ knowledge this is the first time such a comparison has been made for weathered PAH contaminated soils. Considering that most PAH contamination is historic, the optimisation of PAH extraction and the rapid analysis of PAH contaminated soil represents a key requirement for effective environmental and land assessment and management. The different solvents and mixtures compared were hexane [16], [17], dichloromethane [18], [19], [20], acetone: hexane (1:1) [21], acetone: dichloromethane (1:1) [22] and chloroform: methanol (2:1) [23].

## Method details

A brief schematic outlining the steps used for rapid solvent extraction and GC–MS/MS analysis of PAHs in weathered soils are presented in the graphical abstract. More details on the individual steps are provided in the appropriate section below.

### Chemicals

The 16 EPA PAHs analysed in this study were acenaphthene (ACE), acenaphthylene (ACY), anthracene (ANT), benzo(a)anthracene (BAN), benzo(a)pyrene (BAP), benzo(b)fluoranthene (BBF), benzo(g,h,i)perylene (BGP), benzo(k)fluoranthene (BKF), chrysene (CRY), dibenzo(a,h)anthracene (DBA), fluoranthene (FLA), fluorene (FLU), indeno(1,2,3-cd)pyrene (IND), naphthalene (NAP), phenanthrene (PHE) and pyrene (PYR). Gas chromatography grade (99.9% pure) *n*-hexane, dichloromethane and sodium sulphate was obtained from Merck Millipore (Victoria, Australia). Acetone, chloroform and methanol were purchased from Sigma Aldrich (NSW, Australia). A mixture of certified multi-component PAH standards (PAH mix 3) in methanol and methylene chloride (1:1) was purchased from Supelco (Bellefonte, PA). Calibration standards were prepared by diluting the primary PAH standard in hexane. Disposable Teflon coated syringe filter discs (0.2 μm) were provided by Sarstedt (SA, Australia).

### Sample collection, preservation and preparation

• Soil samples were air dried at room temperature one day before use and sifted through a 2 mm sieve to remove large particles before being ground with a pestle and mortar.
• Samples were then sieved through a 600 μm sieve to remove any particles ≥600 μm and then fully homogenized.

### PAH extraction

• Soil heterogeneity in terms of the distribution of PAHs was initially tested following an acetone: hexane extraction to investigate variability in PAH concentrations between replicate soil samples. The results showed that the contaminants were homogenous in distribution within the soils, with replicate soil samples having total PAH concentrations varying by less than 6% (data not shown). PAH extraction from weathered soils was then carried out by using different solvent systems.
• The different solvents systems used were a) hexane, b) dichloromethane, c) acetone: hexane (1:1), d) acetone: dichloromethane (1:1) and e) chloroform: methanol (2:1). Soil moisture was reduced by air drying, preferred over freeze drying which may lead to partial loss of highly volatile PAHs such as NAP. Replicates (n = 3 for each solvent system) of air dried homogenised soil samples were weighed and transferred into 50 mL centrifuge tubes with screw caps containing pre-dried (100 °C) sodium sulphate (5 g) to reduce the moisture content of the soil.
• The mixture was hand-shaken and any lumps were broken using a spatula to ensure homogeneity of soil and sodium sulphate. After mixing, 15 mL of either hexane, dichloromethane (DCM), acetone: hexane (1:1), acetone: DCM (1:1) or chloroform: methanol (2:1) was added to individual tubes. All the tubes were vortexed briefly. The mixtures were further shaken using a mechanical shaker for 10 min at room temperature. All the tubes were centrifuged at 4696*g* for 5 min. Supernatants were transferred into clean 50 mL centrifuge tubes. The remaining pellets were subjected to a second extraction in 10 mL of their respective solvents (breaking up the pellet cake with a spatula, if necessary), employing 5 min shaking time followed by centrifugation. Supernatants from both the extractions were pooled and volumes for each solvent were adjusted to 25 mL with the use of appropriate solvents.
• Pooled mixtures were transferred into fresh 50 mL centrifuge tubes through 0.2 μm Teflon coated filter discs using a syringe. Extracts were centrifuged at 4696*g* for 5 min. An aliquot (50 μL) of each sample was transferred into a 2 mL microcentrifuge tubes together with 950 μL of hexane. The samples were centrifuged at 4696*g* for 5 min and the supernatant was transferred to 2 mL GC vials. Hexane was used as the injection solvent to improve the separation of fractions considering it as a nonpolar solvent, as are PAHs.

### GC–MS/MS analysis

• The identification and quantification of individual PAHs were performed by GC–MS/MS due to the high sensitivity and specificity of this analytical technique for contaminated soil samples. A HP 7890B gas chromatograph (GC) from Agilent Technologies equipped with Agilent PAL autosampler 120 and 10 μL syringe was used for the separation of the PAHs.
• Analytes were separated by a DB—5 ms ultra inert capillary column; comprising a first column of internal diameter 0.25 mm, length 30 m and thickness 0.25 μm and a second column of internal diameter 0.25 mm, length 25 m and thickness 0.25 μm. Analysis involved pulsed splitless mode with an injection volume of 2 μL. The oven temperature was programmed as follows: initial temperature at 50 °C (hold 1 min), then 25 °C min^−1^ to 325 °C, held for 5.2 min. High purity helium gas (>99.999%) was used as carrier gas with a flow rate of 1.4 mL min^−1^ in the first column and 1.5 mL min^−1^ in the second column. Detection of the analytes was performed by employing an Agilent Technologies 7000 c triple quadrupole mass spectrometer (MS) operating in Multiple Reaction Monitoring (MRM) mode. The N_2_ collision cell and He quench gas were set at 1.5 mL min^−1^ and 2.25 mL min^−1^, respectively. The GC–MS inlet temperature was set at 90 °C for 0.01 min, then 900 °C min^−1^ to 325 °C and held at 325 °C until analysis. The transfer line was set at 325 °C. The ion source temperature was set at 350 °C and both quadrupole 1 and quadrupole 2 were set at 205 °C. Quantification of the analytes employed the integrated peak area ratio of the target ion to the internal standard. The PAH analytes were identified based on target ions and retention time order.
• The software used for quantitative analysis was Agilent MassHunter Workstation (version B.07.01/Build 7.1.524.0).

### Quality control

A procedural hexane blank was carried out periodically throughout the study. Blank solutions were passed through the columns with the same protocol as followed for soil PAH extracts. The average blank concentration of three replicates was subtracted from each sample to correct for potential metrological variability. The limit of detection was calculated as three times the standard deviation of the blank. The samples were sent to a NATA accredited external laboratory, ALS Pty Ltd. (Melbourne, Australia) for validation purpose [24].

### Data analysis and validation

Experimental data are presented as means of three independent measurements (three replicate soil samples for each solvent system). Analysis of variance (Single factor ANOVA) was performed on data using the Minitab statistical analysis program. Independent samples *t*-tests were used to compare any significant differences between the different extraction solvents. Mean values were compared using the Least Significant Difference (LSD) test (*P* ≤ 0.05), where the F-value was significant. The standard deviation (SD) are reported in figures, where required.

## Results

**Fig. 1:**
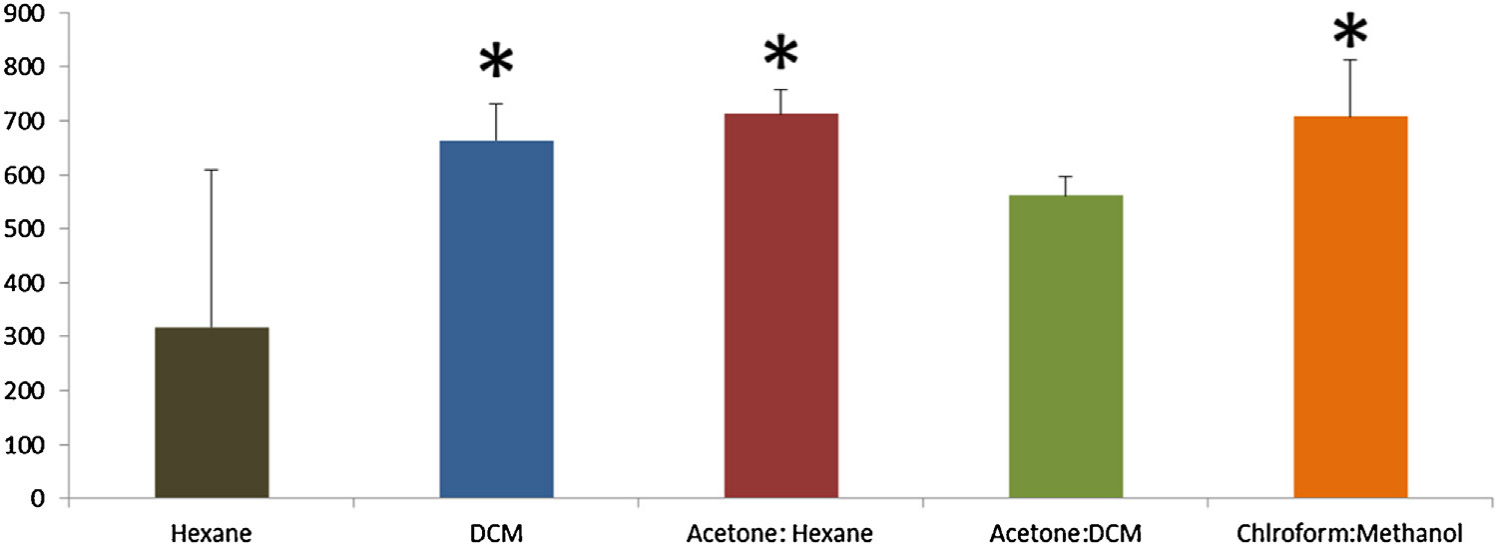
Variation in ΣPAH’s concentrations extracted from weathered soil using different solvents. Error bars show ± standard deviation. Asterisks indicate significantly higher concentrations (p < 0.05). DCM = Dichloromethane.

PAH concentrations (total and individual) recovered from soil samples using the five extraction solvent systems were determined and compared. Having established the homogeneity of the soils in terms of the distribution of PAHs, the efficacy of different solvents to extract PAHs was examined. The total concentrations of PAHs (ΣPAHs) varied considerably between the five different solvent systems (Fig. 1). Highest ΣPAH concentrations, extracted using acetone: hexane, chloroform: methanol and DCM solvent systems were significantly higher than those extracted using hexane and acetone: DCM solvent systems (p < 0.05). The lowest concentration of ΣPAHs were extracted using hexane as a solvent.

The order of the highest concentrations of ΣPAHs extracted from soils using the five solvent systems was as follows: acetone: hexane > chloroform: methanol > DCM > acetone: DCM > hexane. Given the higher toxicities associated with chloroform: methanol and dichloromethane, the use of acetone: hexane provides the best combination of an effective extraction of PAHs from weathered soils coupled to lowest toxicity of the solvents used for extraction.

Further data analysis was carried out to assess the effect of the solvent extraction on the concentration of the 16 USA EPA listed PAHs. The concentration of individual PAHs extracted from weathered soils using different solvents in the present study varied widely with different solvent systems tested (data not shown). —Our results indicate that acetone: hexane may be the choice of extraction solvent for lower ring compounds (2, 3 and 4 ring compounds) and chloroform: methanol the choice of extraction solvent for higher ring compounds (5 and 6 ring compounds) in rapid extraction of PAHs from aged, contaminated soils. A total of 25 mL of the solvent was used in the complete extraction procedure proving it to be environmental friendly when compared with other traditional methods that used large amounts of solvents [12], [25], [26]. The limit of detection for the proposed method was found to be between 2 and 30 ng g^−1^ of soil [27].

**Fig. 2:**
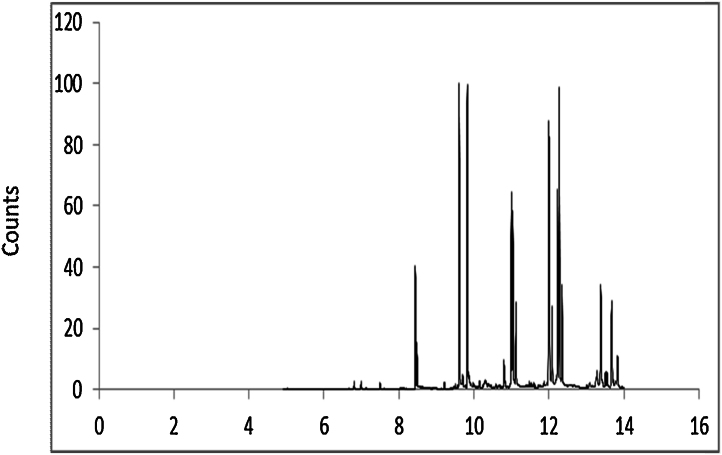
Representative GC- chromatogram of PAH compounds extracted from weathered contaminated soil using acetone: hexane extract. Retention times (min) of the corresponding 16 USEPA PAHs are detailed in the Supplementary Table 1.

Further, this method was compared to values obtained from ALS laboratory GC–MS that utilizes the USEPA method and acetone: DCM (1:1) as the extraction solvent [24]. Statistical analysis of the data showed that there was no significant differences in the ΣPAHs obtained using the current protocol (p =  0.7) indicating that acetone: hexane could be the choice of extraction solvent over acetone: DCM as the latter resulted in extraction of lower concentrations of PAHs during the rapid extraction processes. The 16 EPA priority pollutants were analysed using the obtained chromatographic profiles. The retention times of the 16 PAHs analysed are shown in Supplementary Table 1. The chromatogram presented in Fig. 2 display GC profiles obtained from the PAH contaminated soil using acetone: hexane as the extraction solvent. The peaks of 2- ringed PAHs appeared smallest in all the profiles when compared with other peaks possibly due to their increased volatility compared to higher ring PAHs.

## Conclusion

The extraction protocols developed coupled with GC–MS/MS used in this study allowed us to analyse target compounds at concentrations of nanograms per gram with a high sensitivity and selectivity. Our results showed that acetone: hexane, DCM and chloroform: methanol were effective in the extraction of PAHs while hexane alone yielded the lowest concentrations of PAHs extracted from weathered soil. The simultaneous extraction and clean-up reduced the total analysis time showing it to be more efficient when compared to traditional methods. Considering the hazards of solvent volume and toxicity associated, we suggest that the proposed method using acetone: hexane can be considered “greener” than the existing ones for the analysis of PAHs [24]. It is crucial that management decisions made on the basis of the levels of PAHs detected are based on the maximum levels of PAHs extracted.
