# Supplementary material for: Comparison of rapid solvent extraction systems for the GC–MS/MS characterization of polycyclic aromatic hydrocarbons in aged, contaminated soil
Source: MethodsX. 2016 Apr 26;3:364–70. doi: 10.1016/j.mex.2016.04.007 (PMC4864413; doi:10.1016/j.mex.2016.04.007)
Supplement: Supplementary file 1 [file mmc1.doc]

Supplementary Table 1: Retention times of the 16 PAH’s (US EPA priority pollutants) analysed in this study.

| PAH  ( µg g¯^1^ ) | Retention time (minutes) | PAH  ( µg g¯^1^ ) | Retention time (minutes) |
| --- | --- | --- | --- |
| NAP | 5.016 | BAN | 11.004 |
| ACY | 6.807 | CRY | 11.043 |
| ACE | 6.996 | BBF | 12.00 |
| FLU | 7.502 | BKF | 12.02 |
| PHE | 8.442 | BAP | 12.282 |
| ANT | 8.486 | IND | 13.389 |
| FLA | 9.614 | DBA | 13.402 |
| PYR | 9.83 | BGP | 13.679 |
